# Engineering an L-cell line that expresses insulin under the control of the glucagon-like peptide-1 promoter for diabetes treatment

**DOI:** 10.1186/1472-6750-11-99

**Published:** 2011-11-03

**Authors:** Mina Rasouli, Zalinah Ahmad, Abdul Rahman Omar, Zeenathul N Allaudin

**Affiliations:** 1 Laboratory of Vaccines and Immunotherapeutics, Institute of Bioscience, Universiti Putra Malaysia, UPM Serdang, Selangor, Malaysia; 2 Department of Pathology, Faculty of Medicine and Health Sciences, Universiti Putra Malaysia, UPM Serdang, Selangor, Malaysia; 3 Department of Veterinary Pathology & Microbiology, Faculty of veterinary medicine, Universiti Putra Malaysia, UPM Serdang, Selangor, Malaysia

## Abstract

**Background:**

Diabetes mellitus is a complicated disease with a pathophysiology that includes hyperinsulinemia, hyperglycemia and other metabolic impairments leading to many clinical complications. It is necessary to develop appropriate treatments to manage the disease and reduce possible acute and chronic side effects. The advent of gene therapy has generated excitement in the medical world for the possible application of gene therapy in the treatment of diabetes. The glucagon-like peptide-1 (GLP-1) promoter, which is recognised by gut L-cells, is an appealing candidate for gene therapy purposes. The specific properties of L-cells suggest that L-cells and the GLP-1 promoter would be useful for diabetes therapy approaches.

**Results:**

In this study, L-cells were isolated from a primary intestinal cell line to create suitable target cells for insulin expression studies. The isolated cells displayed L-cell properties and were therefore used as an L-cell surrogate. Next, the isolated L-cells were transfected with the recombinant plasmid consisting of an insulin gene located downstream of the GLP-1 promoter. The secretion tests revealed that an increase in glucose concentration from 5 mM to 25 mM induced insulin gene expression in the L-cells by 2.7-fold. Furthermore, L-cells quickly responded to the glucose stimulation; the amount of insulin protein increased 2-fold in the first 30 minutes and then reached a plateau after 90 minutes.

**Conclusion:**

Our data showed that L-cells efficiently produced the mature insulin protein. In addition, the insulin protein secretion was positively regulated with glucose induction. In conclusion, GLP-1 promoter and L-cell could be potential candidates for diabetes gene therapy agents.

## Background

Diabetes mellitus is characterised by metabolic disorders and abnormally high blood glucose, which are caused by the destruction of the β-cells of the pancreas, insulin resistance and/or insulin deficiency. Achieving a normal circulating glucose level is a major goal for therapeutic intervention in diabetes patients. However, the current standard of care, which consists of constant monitoring and precise insulin loading through injections, puts patients at risk for acute diabetes complications [1]. Gene therapy can be a successful treatment for diabetes if insulin can be produced through a glucose-regulated pathway and if the insulin thus produced can elicit responses to glucose fluctuation levels that are similar to those induced by natural insulin secretion. Furthermore, candidate cells for gene therapy need to express enzymes for post-translational processing of pro-insulin into mature insulin. Some research groups have genetically modified several cell types to produce functional insulin [2,3]. However, their studies showed that the engineered cells could not produce satisfactory insulin substitutes. This is because the cell types used do not possess all the essential properties that would mimic the natural physiological regulation of insulin secretion.

Enteroendocrine cells, which are located in the gut lumen, secrete incretin hormones such as glucagon-like peptide-1 (GLP-1, from L-cells) and glucose-dependent insulinotropic polypeptide (GIP, from K-cells) that act on pancreatic β-cells to stimulate the release of insulin. The specific factors that regulate GLP-1 and GIP secretion are very similar to those that regulate insulin secretion by β-cells [4]. In addition, L- and K-cells express carboxypeptidase H and pro-hormone convertases 2 and 3, the same processing enzymes used by β-cells to process mature insulin [5]. Certain properties of enteroendocrine cells, including glucose sensitivity, insulin processing capability and a regulated secretion pathway, make them ideal potential candidate cells for diabetes gene therapy.

Previous studies reported that genetically engineered K-cells expressed insulin protein under the control of the GIP promoter [6,7]. Additionally, other studies showed that a transgenic mouse expressing a recombinant insulin gene under the control of the GIP promoter was capable of normalising blood glucose levels in response to an increase in glucose consumption [6]. Furthermore, recent studies have revealed that engineered L-cells produced insulin protein as a result of various stimuli. These results prove that L-cells contain the required factors to synthesise, process and secrete mature insulin [8]. However in these experiments, universal promoters (such as viral promoters) were employed to introduce the insulin gene into the L-cells [9]. Thus, caution has to be exercised because viral promoters are not cell specific and the genes they carry could therefore be expressed in all types of cells.

GLP-1 is one of the products of the proglucagon gene, which expresses a number of different hormones in different tissues. When glucagon is produced in the α-cells of the pancreas; glicentin, GLP-I and II are expressed in the L-cells of the intestine [10]. Extensive research has led to the identification of a promoter region that mediates cell-specific gene transcription in each tissue. It was reported that approximately 2.3 kb of the proglucagon gene 5'-flanking sequences control highly tissue-specific gene transcription of GLP-1 in the L-cells [11]. The unique properties of L-cells and the GLP-1 promoter provide a strong rationale to use the GLP-1 promoter to express glucose-regulated insulin in intestinal L-cells for the potential treatment of diabetes. In this study, the region of the proglucagon promoter that is recognised by L-cells was utilised in expressing the insulin gene in vitro. We studied the ability of the engineered L-cells to produce insulin under different glucose concentrations and at various time points. For these purposes, we extracted L-cells from intestinal endocrine cells [12]. The extracted cells are useful for the cell-based study of intestinal L-cells, specifically in the investigations of its potential as a gene-therapy candidate for diabetes treatment.

## Methods

### Plasmid construction

**Figure 1:**
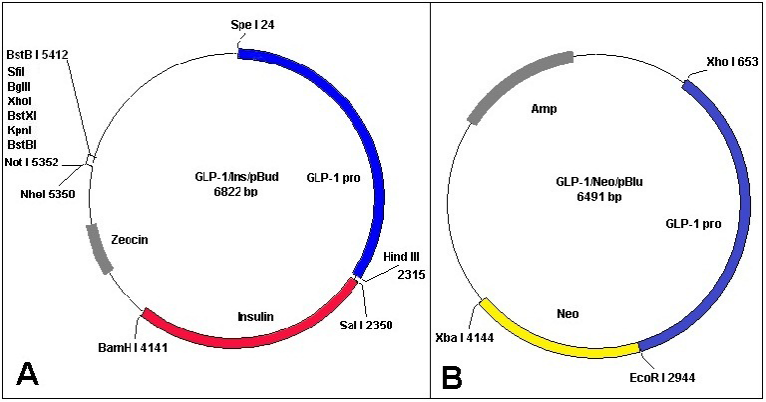
**Construction and restriction maps of recombinant plasmids**. (A) Schematic diagram of GLP-1/Ins/pBud plasmid: GLP-1 promoter (GLP-1 pro, blue part) and insulin gene (red part) in the pBudCE4.1 vector. The pBudCE4.1 vector contains the zeocin resistant gene. (B) Schematic diagram of GLP-1/Neo/pBlu plasmid: GLP-1 promoter (GLP-1 pro, blue part) and neomycin resistant gene (Neo) in the pBluescript-II-SK vector. The pBluescript-II-SK vector contains the ampicillin (Amp) resistant gene.

To construct the plasmid, the proglucagon promoter region (approximately 2,300 bp) was amplified from the Glu.BS plasmid, which was kindly provided by Dr. Yvan Gosmain from the University of Geneva [13]. In addition, a human insulin gene (approximately 1,800 bp) was obtained from human genomic DNA. Furthermore, approximately 1,200 bp of a neomycin resistant gene was amplified from pcDNA_3 _plasmid (Invitrogen, USA). To produce GLP-1/Ins/pBud plasmid, the proglucagon promoter fragment and insulin gene were inserted into the pBudCE4.1 vector (Invitrogen, USA). Similarly, the proglucagon promoter and neomycin resistant gene were cloned into a pBluescript-II-SK vector (Stratagene, USA) to create a GLP-1/Neo/pBlu plasmid (Figure 1). The sequences of primers and the positions of the restriction enzymes used are illustrated in table 1. All the enzymes and kits were purchased from Fermentas, Lithuania.

**Table 1:** The primers sequences for PCR amplification and related restriction enzymes involved

Name	Sequences
**LP.Bud-F**	**5' **AT GAG AAA GCT TGT AGA CAG GTG GAG **3'**
	*Hind III*
	
**LP.Bud-R**	**5' **AC AAC ACT AGT GCT TCC AGT CAA ACC **3'**
	*Spe I*

**Ins-F**	**5' **AA GTT GTC GAC AGG CTG CAT CAG AAG **3'**
	*Sal I*
	
**Ins-R**	**5' **A TAG GAT CCA CAG GGA CTC CAT CAG **3'**
	*Bam H I*

**LP.Blu-F**	**5' **G AAT TCG AGC TGA GAG GAG GTG TAG **3'**
	*EcoRI*
	
**LP.Blu-R**	**5' **C TCG AGA TAC CTG CCT ACC ACT GTC **3'**
	*XhoI*

**No-F**	**5' **GA ATT CCA GAA GTA GTG AGG AGG **3'**
	*EcoR I*
	
**No-R**	**5' **T CTA GAT ACA TTG ATG AGT TTG GAC **3'**
	*Xba I*

LP.Bud-F and LP.Bud-R are the primers used for amplification of GLP-1 promoter. *Hind III *and *Spe I *are the restriction enzymes used to facilitate GLP-1 promoter cloning into the pBudCE4.1 vector. LP.Blu-F and LP.Blu-R are the primers for amplification of GLP-1 promoter. *EcoR I *and *Xho I *are the restriction enzymes used to facilitate GLP-1 promoter cloning into the pBluescript II SK vector. Ins-F and Ins-R are the primers for amplification of insulin gene. *Sal I *and *BamH I *are restriction enzymes used to facilitate the cloning of insulin gene into the pBudCE4.1 vector. No-F and No-R are the primers for amplification of neomycin resistant gene. *EcoR I *and *Xba I *are the restriction enzymes used to facilitate the cloning of neomycin resistance gene into the pBluescript II SK vector. Underlined sequences are the restriction enzyme sites.

### Cells and transfection

The STC-1 cell line that was derived from an endocrine tumour of the murine intestine was kindly provided by Prof. Douglas Hanahan from the University of California. The STC-1 cells were cultured in DMEM (Dulbecco's Modified Eagle's Medium; PAA, Austria) containing 10% FBS (Fetal bovine serum; PAA, Austria). Cells were transfected with the GLP-1/Ins/pBud plasmid or GLP-1/Neo/pBlu plasmid using Lipofectamine 2000 Transfection Reagent (Invitrogen, USA). After 48 hrs, the cells transfected with GLP-1/Ins/pBud or GLP-1/Neo/pBlu plasmid were treated with zeocin (Invitrogen, USA) or geneticin antibiotics (Sigma, USA), respectively. The media containing antibiotic was replaced every three days for at least two weeks, until individual clones could be selected. Five stable clones were isolated for further studies.

MTT assay was employed to assess the cytotoxicity of the zeocin and geneticin antibiotics. This assay determines the appropriate amount of antibiotic sufficient to kill all cells without the cells developing antibiotic resistant properties. Initially, STC-1 cells (without any antibiotic resistant) were treated with different concentrations of either zeocin or geneticin in the range of 0 to 1 mg/ml for two weeks. Subsequently, cells were incubated with MTT (3-(4, 5-dimethylthiozol-2-yl)-3, 5-dipheryl tetrazolium bromide) (Sigma, USA) for 5 hrs in 37°C followed by the addition of DMSO (Dimethyl sulfoxide; Sigma, USA) for 30 min. Finally, optical density of the solutions was read at 560 nm using an ELISA plate reader.

### Secretion test

Three million of each of the five isolated clones were grown for two days prior to the experiment. The secretion test was performed as previously described [6,14]. Briefly, the cells were incubated in basal media (5 mM glucose, 1% FBS) overnight. On the day of the experiment, the media was changed to fresh basal media. After two hrs, the cells were divided into two groups; one group was exposed to media containing 5 mM glucose and the second group received media supplemented with 25 mM glucose for one hour. Finally, the media and the cells were collected separately for insulin expression studies.

### RT-PCR and quantitative-PCR

Total RNA was extracted from the five isolated clones and the STC-1 cells using Qiagen RNA extraction kit (QIAGEN, Germany). The cDNA was synthesised by an iScript cDNA Synthesis Kit (Bio-Rad, CA); PCR was then conducted using the specific primers for the target genes (Table 2). Mouse β-actin was amplified as a positive control. Q-PCR was performed on the synthesized cDNA of STC-1 and five isolated clones using an EvaGreen PCR Kit (Bio-Rad, CA) and a thermal cycler from Bio-Rad, CA. The data were analysed by CFX Manager Software (version 1.0.1035.131, Bio-Rad, CA). The mouse β-actin and β-2 microglobulin values were employed for normalisation of the Q-PCR data.

**Table 2:** The primers sequences for RT-PCR and quantitative-PCR assays

Name	Sequences
**GLP-rt-F**	**5' **GGC ACA TTC ACC AGC GAC TAC **3'**
	
**GLP-rt-R**	**5' **CA ATG GCG ACT TCT TCT GGG **3'**

**In-rt-F**	**5' **A ACG AGG CTT CTT CTA CAC ACC **3'**
	
**In-rt-R**	**5' **TTC CAC AAT GCC ACG CTT CTG **3'**

**Ac-rt-F**	**5' **GTG TGA TGG TGG GAA TGG GTC **3'**
	
**Ac-rt-R**	**5' **AG GAA GAG GAT GCG GCA GTG **3'**

**B2m-rt-F**	**5' **CTG GTC TTT CTG GTG CTT GTC **3'**
	
**B2m-rt-R**	**5' **AT GTG AGG CGG GTG GAA CTG **3'**

GLP-rt-F and GLP-rt-R were used to amplify mouse GLP-1 mRNA. In-rt-F and In-rt-R were used to amplify human insulin mRNA. Ac-rt-F and Ac-rt-R were used to amplify mouse β-actin mRNA. B2m-rt-F and B2m-rt-R were used to amplify mouse β-2 microglobulin (β2 m) mRNA.

### ELISA

The insulin secreted into the culture media was determined using the ultrasensitive human insulin ELISA kit (ALPCO, USA). This ELISA kit has 100% cross reactivity with mature human insulin only. The samples for the ELISA experiment were collected from the media during the secretion test in different glucose concentrations and at different time points.

### Western blotting

A native polyacrylamide gel electrophoresis (native-PAGE) was prepared as previously described [15]. The total proteins from the cells were separated on the gel and then transferred onto a nitrocellulose membrane (Whatman, UK) using a semi-dry system. The presence of human insulin was detected by incubation of the membrane with a 1:1000 dilution of the primary antibody, a mouse monoclonal antibody against human insulin (Abcam, UK), followed by incubation in a 1:10,000 dilution of the secondary antibody, a rabbit polyclonal antibody against mouse IgG conjugated to alkaline phosphatase (Abcam, UK). Finally, proteins were visualised using a BCIP-NBT kit (Nacalai Tesque, Japan). The 5-bromo-4-chloro-3'-indolyphosphate (BCIP) and the nitro-blue tetrazolium (NBT) react with alkaline phosphatase to produce a purple compound which indicates the presence of protein.

### Immunocytochemistry

Transfected cells were seeded on a cover-slip two days prior to the experiment. After the fixation and permeabilisation stages, the cells were incubated with the mouse monoclonal antibody against human insulin (1:200). The cells were then exposed to goat polyclonal antibody against mouse IgG conjugated to FITC (Abcam, UK; 1:1000). Finally, a 1 μg/ml DAPI solution (4,6-diamidino-2-phenylindole; Sigma, USA) was used to dye the nuclei and then stained cells were imaged by a fluorescence microscope.

## Results

### Isolation of L-cells

**Figure 2:**
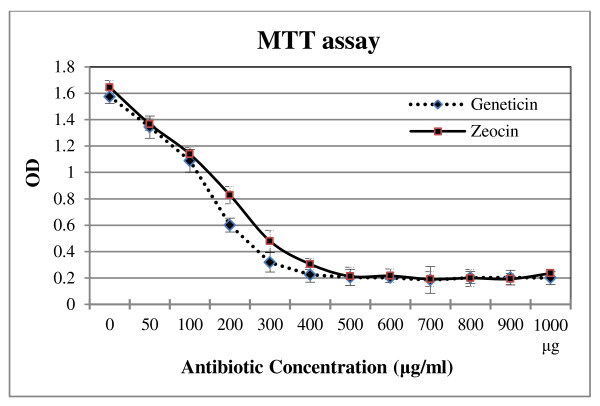
**An MTT assay was used to check geneticin and zeocin cytotoxicity level**. The viability of STC-1 cells was studied in the presence of 0 to 1 mg/ml geneticin (diamond) and zeocin (square) antibiotics. 400 μg/ml of geneticin and 500 μg/ml of zeocin were the lethal concentrations that kill all the STC-1 cells. Error bars indicate standard deviation, p < 0.05.

As a basic model for L-cells, GLP-1 -secreting cell line was generated using the GLP-1/Neo/pBlu plasmid, which was manipulated in such a manner that the GLP-1 promoter was located upstream of the neomycin resistant gene (Figure 1-B). It was reported previously that ~5% of STC-1 cells produce GLP-1, whereas no immunoreactivity was detected with insulin antibodies [12]. Because the STC-1 cell line is a suitable source of intestinal cells, it was used for isolation of the L-cells. The STC-1 cells were transfected with GLP-1/Neo/pBlu and then treated with 400 μg/ml of geneticin antibiotic, which was measured using an MTT assay (Figure 2). After transfection, the cells that received and recognised the GLP-1 promoter were able to express the neomycin resistant protein and survive under the geneticin antibiotic treatment. The first five stable clones were isolated and then propagated for further analysis.

### GLP-1 secretion assay from stable transfected cells

**Figure 3:**
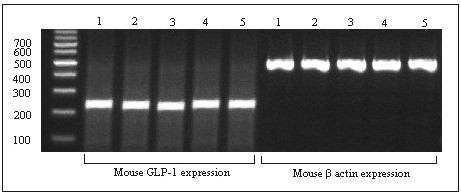
**The results of RT-PCR analysis of expression of mouse GLP-1 and mouse β-actin mRNA**. The five isolated clones from the STC-1 cell line (1-5) were subjected to RT-PCR. The PCR products of GLP-1 and β-actin were 250 bp and 578 bp, respectively, when compared with a 100-bp DNA ladder. All the five clones express mouse GLP-1 mRNA as well as mouse β- actin.

**Figure 4:**
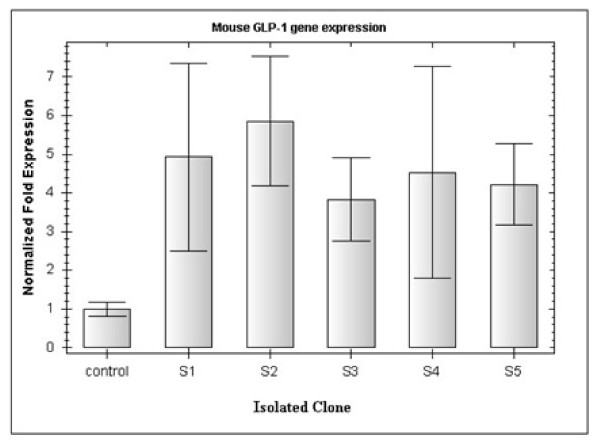
**Analysis of GLP-1 mRNA expressions using quantitative-PCR**. GLP-1 expressions in five isolated L-cells and STC-1 cells (as a control) were measured. All the isolated cells expressed more GLP-1 mRNA than the control cells. The BioRad CFX Manager software was employed to analyse GLP-1 expression data. The mouse β-actin and β-2 microglobulin (β2 m) mRNA levels were used to normalize the values. Error bars indicate standard deviation, p < 0.05.

The GLP-1 gene is expressed specifically in L-cells therefore the presence of its mRNA authenticates the identity of the isolated transfected cells. The RT-PCR products of mouse β-actin (578 bp) and mouse GLP-1 (250 bp) mRNA for the five isolated clones were examined on an agarose gel. As shown in Figure 3, all the five clones expressed mouse GLP-1 mRNA, thus confirming that all the isolated cells were L-cells. Furthermore, because only ~5% of STC-1 cells produce GLP-1, it was expected that the isolated L-cells would express more GLP-1 mRNA than primary cells. Therefore, the GLP-1 expression level of the five isolated clones as well as of the STC-1 cells (as control) was studied by Q-PCR (Figure 4). Although the same number of cultured cells and conditions were used, there was a significant difference in the GLP-1 expression level of the isolated clones and the primary cells, whereas only small differences in mRNA levels among the five isolated clones were observed. The GLP-1 mRNA expression in clone 2 (L-2) was about 5.8-fold that of the control. Therefore, the L-2 clone with the highest GLP-1 expression level was used for subsequent studies.

### Recombinant insulin transfection of L-cell line

To create an insulin-expressing L-cell line, a pBudCE4.1 vector with a zeocin resistance gene was selected, allowing the use of one marker to select L-cells (geneticin) and one to select insulin-producing cells (zeocin). The reason for using this vector was because the isolated L-cells have already been resistant to the neomycin antibiotic (geneticin) when they were transfected by GLP-1/Neo/pBlu plasmid. Therefore, a different vector with different selectable marker was needed to isolate insulin expression cell line from the previously isolated L-cells. The pBudCE4.1 is a co-expression vector with CMV and EF-1-α promoters. Because the aim of the project was to study the ability of the GLP-1 promoter to express the insulin gene, the EF-1-α promoter was omitted and the CMV promoter was replaced with GLP-1 promoter. Finally, the human insulin gene was sub-cloned downstream of the GLP-1 promoter in the engineered pBudCE4.1 vector (Figure 1-A). The L-2 cell line was transfected by the GLP-1/Ins/pBud plasmid and then grown in media containing 500 μg/ml of zeocin antibiotic; this concentration was determined to be appropriate by using an MTT assay (Figure 2). The first five clones that appeared were selected and grown for further insulin expression analyses.

### Secretion of insulin from L-cell line

**Figure 5:**
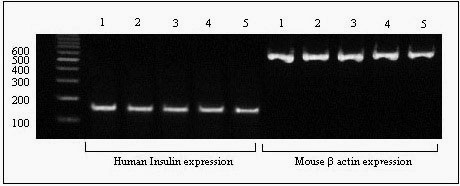
**The results of RT-PCR analysis of expressions of human insulin and mouse β-actin mRNA**. Five stable clones in the antibiotic condition were analysed for insulin gene expression. The PCR products of the insulin and β-actin genes were 150 bp and 578 bp, respectively, when compared with a 100-bp DNA-ladder. All the five clones express human insulin mRNA as well as mouse β- actin.

**Figure 6:**
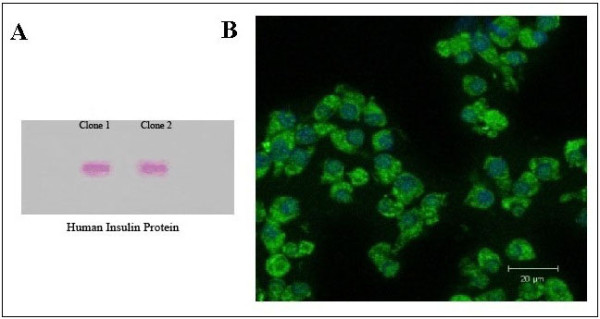
**The expression of human insulin protein was analysed using western blotting and immunocytochemistry assays**. (A) The result of western blotting shows that mature human insulin was secreted by engineered L-cells. Total protein was extracted from L-1 and L-2. (B) Immunocytochemistry assay confirmed insulin expression in the L-cells. The green sections show that the cytoplasm of the cells expresses human insulin, and the blue sections are nuclei stained by DAPI.

The expression of insulin mRNA was studied by RT-PCR using special primers for human insulin mRNA (150 bp) and using mouse β-actin mRNA (578 bp) as a positive control. The resulting PCR products indicated that all five selected cells successfully expressed human insulin mRNA (Figure 5). The mature and active insulin protein has a molecular mass of approximately 5.800 kDa and includes two polypeptide chains (A- and B-chain) that are linked by a disulfide bond [16]. A specific antibody that reacts only with the intact insulin molecule (not A-chain, B-chain or pro-insulin) was used to detect the accurate assembly of the human insulin protein. The routine PAGE method utilises a reducing buffer such as SDS and DTT [17]. Because reducing conditions dissociate the A- and B-chain of the insulin protein, native-PAGE was employed for insulin analysis. Furthermore, the SDS-PAGE method is preferable for the optimal separation of proteins > 30 kDa [17]; therefore, for the small insulin protein, native-PAGE is strongly recommended [15]. The total proteins extracted from L-1 and L-2 were analysed by western blotting. As observed in Figure 6-A, two samples produced human insulin, confirmed by analysis of the size and structure of the protein secreted. Additionally, an immunocytochemical assay exhibited the existence of human insulin inside the transfected L-cells (Figure 6-B).

**Figure 7:**
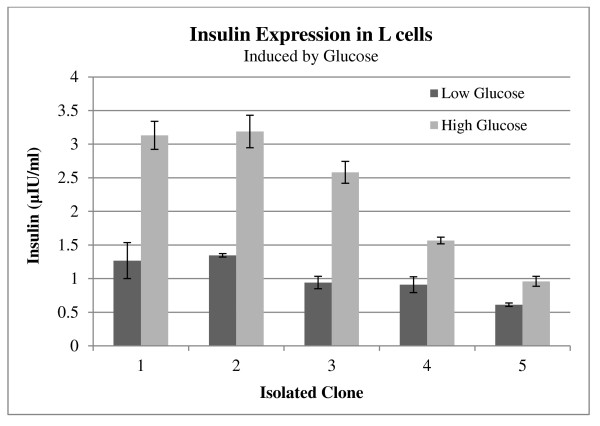
**Glucose responsive insulin secretion was analysed in L-cells**. Insulin expressions in five isolated cells were studied by ELISA. The samples were collected during secretion tests. Insulin expression in all the isolated clones increased significantly with 25 mM glucose induction. Error bars indicate standard deviation, one-way ANOVA with unequal variances p < 0.05.

**Figure 8:**
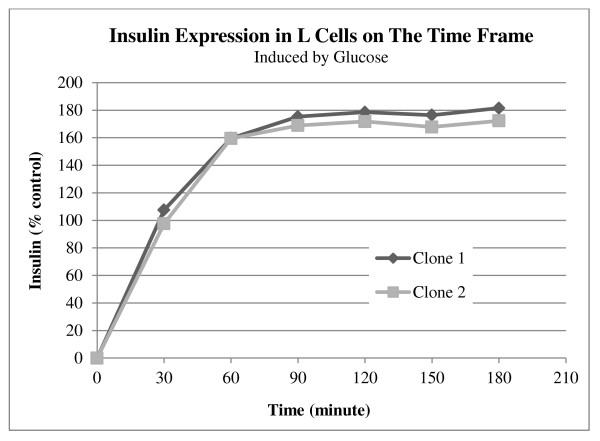
**The relationship between the insulin secretion and time was investigated by ELISA**. Clone number 1 and 2 were used for this experiment. The samples was collected every 30 min after inducing with glucose. The values were normalized to the amount of insulin secreted under basal conditions.

The insulin mRNA level was measured by Q-PCR to verify the effects of glucose concentration on mRNA synthesis. Although the human insulin mRNA content in high-glucose media was greater than in the low-glucose concentration, statistical analyses showed that this increase was not significant (data not shown). The amount of insulin secreted in response to two different glucose concentrations was quantified by ELISA (Figure 7). Insulin levels in the five isolated clones were significantly increased when the cells were treated with high glucose media (n = 4, p < 0.005). In 5 mM glucose the highest insulin level was 1.3 μIU/ml per well (L-2), whereas the lowest was 0.69 μIU/ml (L-5). Samples L-1 and L-2 had the highest insulin concentration levels in 25 mM glucose, approximately 3.13 μIU/ml and 3.18 μIU/ml, respectively. To study the relationship of insulin expression with time, the insulin level was measured at six time points over a period of three hrs. L-1 and L-2, which showed the highest insulin secretion levels, were employed for determination of the speed of insulin secretion in L-cells. Figure 8 shows a rapid increase of insulin in the first 30 min after the addition of the media containing 25 mM glucose. After that, there was a slow but steady rise in the insulin secretion before reaching a plateau at 90 min.

## Discussion

Although there are some enteroendocrine cell lines available [18], the necessity for pure cells to study GLP-1 secretion motivated the extraction of L-cells from an intestinal cell line. Several research groups have employed an STC-1 cell line to create insulin expression cells as surrogate β-cells [14,19]. In 2000, Cheung et al. successfully expressed an insulin gene in STC-1 cells using a GIP promoter. Their results initiated efforts to engineer endocrine cells for diabetes treatment [6]. Furthermore, Ramshur et al. (2002) produced insulin/GIP co-producing cells that secreted insulin and a GIP protein in response to GIP secretagogues. In this work, L-cells were successfully isolated from the STC-1 cell line using the same approach as Ramshur et al. used [7].

We designed a GLP-1/Neo/pBlu plasmid that was specifically recognised by L-cells, providing an opportunity to isolate L-cells through antibiotic resistance of transfected cells. The L-cells showed high levels of the GLP-1 mRNA in comparison with primary STC-1 cells, suggesting that these isolated cells are suitable model for enteroendocrine L-cells related studies. The Q-PCR results showed that the levels of GLP-1 mRNA among the five isolated clones were slightly different. This may be due to the ability of different cells to express related genes or the comparative purity of the isolated cells. The cells extracted by this method are not only useful for studies of insulin expression specifically for diabetes gene therapy, but they also have potential to become a model for L-cell line physiology and activity studies in vitro.

To study insulin expression in the L-cells, we constructed a new plasmid that includes a human insulin gene under the control of the GLP-1 promoter. This construct could be especially useful for the production of insulin in L-cells because the pro-glucagon promoter is cell-specific. Some research groups have studied insulin expression in the L-cell line using a viral promoter and showed that L-cells were able to produce and release insulin protein [9,20]. Our results herein also demonstrate that L-cells can reliably process insulin production, where successfully expression of insulin was shown in the isolated L-cells by western blotting and immunocytochemistry test. However, in our project the pro-glucagon promoter was utilised to control insulin gene expression. This unique feature is distinctive and differentiates our work from the previous ones. In this study, the insulin producing L-cell line was created by transfecting isolated L-cells with the manipulated construct (GLP-1/Ins/pBud) and then selecting the cells with zeocin antibiotic.

Previous studies have revealed that L-cells are glucose sensitive and respond to glucose changes efficiently. Reimann et al. (2002) showed that 25 mmol/l of glucose enhanced GLP-1 secretion up to 3.4-fold compared to 0.5 mmol/l [21]. In addition, Gribble et al. (2003) showed that a 5 mM glucose concentration increased GLP-1 secretion and further studied the glucose sensitivity mechanism in L-cells [22]. Our quantitative gene expression analysis revealed that a 25 mM glucose concentration increased insulin secretion up to 2.7-fold (L-3). Although, the amount of insulin secretion was not identical in the five isolated clones, they effectively responded to the change in glucose concentration. On the other hand, in our study the highest insulin secretion was 3.189 μIU/ml from L-2 in the presence of 25 mM glucose, which was lower than the previous reports [9,20]. The different expression levels may be due to the use of different promoters. In the previous reports, the viral promoter was employed to study the expression of insulin in L-cells instead. Viral promoters can transcript a high copy number of their downstream gene and express genes in all types of cells. These viral features increase the amount of gene expression that is controlled by the viral promoters.

Q-PCR analysis showed that the transcription expression level of the insulin gene was not a function of glucose concentration, as no increase in mRNA synthesis was observed in response to increased level of glucose in the media. This view is supported with the previous studies on the expression of insulin using the GIP promoter, where the mRNA expression level of insulin was not glucose-regulated [7,14]. For a gene therapy approach, a quick response to nutrient changes is one of the crucial requirements of an insulin-producing cell line. Reimann et al. (2004) showed that L-cells have the ability to respond to stimulators quickly [23]. We also determined the speed of insulin secretion into the cell culture media after adding 25 mM glucose. The result showed that the amount of insulin doubled in the first 30 min but reached a plateau after 90 min. This property may be useful for insulin gene therapy because L-cells are able to respond to fluctuating glucose concentrations in a short amount of time.

## Conclusions

In conclusion, we established a murine L-cell line that expressed human insulin under the control of the GLP-1 promoter. Our data showed that insulin expression and secretion increased in response to the change in glucose concentration and also that L-cells efficiently produced mature insulin protein. Our results revealed that the GLP-1 promoter and L-cells can be appropriate candidates for diabetes gene therapy. However, it is very important that this construct is studied in vivo to verify its performance in natural situations.

## List of abbreviations

GLP-1: glucagon-like peptide-1; GIP: glucose-dependent insulinotropic polypeptide; DMEM: dulbecco's modified eagle's medium; FBS: fetal bovine serum; RT-PCR: reverse transcriptase- polymerase chain reaction; Q-PCR: quantitative-PCR; MTT: 3-(4,5-Dimethylthiazol-2-Yl)-2,5-Diphenyltetrazolium Bromide; DMSO: Dimethyl sulfoxide; ELISA: enzyme-linked immunosorbent assay; native-PAGE: native polyacrylamide gel electrophoresis; SDS-PAGE: sodium dodecyl sulfate polyacrylamide gel electrophoresis; 5-bromo-4-chloro-3'-indolyphosphate: BCIP; nitro-blue tetrazolium: NBT; FITC: fluorescein isothiocyanate; DAPI: 4,6-diamidino-2-phenylindole; CMV: cytomegalovirus; EF-1-α: elongation factor 1 alpha.

## Competing interests

The authors declare that they have no competing interests.

## Authors' contributions

MR: participated in the design of the project, carried out the laboratory works, and drafted the manuscript. ZA: lead the project, participated in the design of the project, helped to draft and edit the manuscript. ARO: participated in the design of the project. ZNA: participated in design of project. All authors have read and approved the final manuscript.
